# Application of CRISPR Cas Systems for Biosensing

**DOI:** 10.3390/bios13070672

**Published:** 2023-06-23

**Authors:** Chung Chiun Liu, Yifan Dai

**Affiliations:** Department of Chemical and Biomolecular Engineering, Case Western Reserve University, Cleveland, OH 44106, USA; yxd176@case.edu

The essential properties of a biosensor are its sensitivity and selectivity to detect, monitor and quantify the biomarker(s) for the interests of medicine. Bio-recognition mechanism is the core element of a biosensor. New designs and methods for advancing the sensitivity and selectivity of a bio-recognition mechanism of a biosensor are scientifically and practically relevant and significant.

A conventional bio-recognition element, such as an antibody, realizes its sensing function through molecular interactions. The downstream transduction of this sensing signal relies on externally designed strategies, such as surface impedance or light scattering [1]. In contrast, CRISPR (Clustered Regularly Interspaced Short Palindromic Repeats) is a programmable gene editing tool that provides the capability to realize targeted cleavage of nucleic acids [2], thereby the nature of CRISPR Cas systems is collective machinery for sensing and actuating [3]. This advantage of CRISPR Cas systems allows engineers to design simple interfaces and molecular circuits to utilize the programmability of CRISPR to detect a wide range of targets of interest, from proteins to nucleic acids [4,5,6,7,8,9].

This Special Issue, “Application of CRISPR (Clustered Regularly Interspaced Short Palindromic Repeats) Cas Systems for Biosensing”, intends to bring awareness to and demonstrate the uniqueness of the CRISPR-Cas systems for advancing biosensing. It is hoped that the advancement of CRISPR-Cas based bio-recognition mechanism will lead to a biosensor system with excellent and improved sensitivity and selectivity. Specifically, the methods and the techniques of integrating CRISPR-Cas into various biosensing systems, the design strategies of a CRISPR-Cas system in a biosensor, and the integration between conventional nucleic acid probe-based recognition elements and CRISPR-based recognition elements require additional research and development endeavors. Thus, expanding scientific research and development efforts by biosensor researchers for CRISPR-Cas-based biosensing systems are timely and important to translational biomedical science. Different CRISPR-Cas biosensing systems are described in the manuscripts of this Special Issue, demonstrating the applicability of a CRISPR-Cas system in biosensing. Furthermore, various methods and techniques in cooperating the CRISPR-Cas system into a completed biosensor system are discussed and presented. The research discussed in this Special Issue exhibits the utilization of a CRISPR-Cas biosensing system for detecting different biomarkers and/or measuring a special need.

Specifically, in this issue, Arold S. et al. described a systematic guideline on selecting the right CRISPR systems for in vitro application [10], which provides a tutorial for researchers to design and engineer CRISPR for diverse in vitro applications. To understand the performance of CRISPR-Cas 9 on genome engineering [11], Ozsoz M. et al. developed a carbon nanotube-based electrochemical genosensor capable of detecting mutations introduced by Cas9. Liang, D. et al. designed a Cas12a-based lateral flow assay to detect spinal muscular atrophy [12]. Liang, D. et al. further demonstrated using Cas14a to detect the same disease [13]. Liang, Q. et al. interfaced optogenetic control and CRISPR to engineer a novel approach to modulate metabolic burden [14]. These works showcase the diverse capabilities of CRISPR-Cas-based systems for engineering and medicine.
